# Genetic Structures of Copy Number Variants Revealed by Genotyping Single Sperm

**DOI:** 10.1371/journal.pone.0005236

**Published:** 2009-04-22

**Authors:** Minjie Luo, Xiangfeng Cui, David Fredman, Anthony J. Brookes, Marco A. Azaro, Danielle M. Greenawalt, Guohong Hu, Hui-Yun Wang, Irina V. Tereshchenko, Yong Lin, Yue Shentu, Richeng Gao, Li Shen, Honghua Li

**Affiliations:** 1 Department of Molecular Genetics, Microbiology, and Immunology/The Cancer Institute of New Jersey, University of Medicine and Dentistry of New Jersey Robert Wood Johnson Medical School, Piscataway, New Jersey, United States of America; 2 Bergen Center for Computational Science, University of Bergen, Bergen, Norway; 3 Department of Genetics, University of Leicester, Leicester, United Kingdom; 4 Department of Biometry, University of Medicine and Dentistry of New Jersey Robert Wood Johnson Medical School, Piscataway, New Jersey, United States of America; 5 Department of Statistics, Rutgers University, Hill Center for the Mathematical Sciences, Piscataway, New Jersey, United States of America; University of Montreal, Canada

## Abstract

**Background:**

Copy number variants (CNVs) occupy a significant portion of the human genome and may have important roles in meiotic recombination, human genome evolution and gene expression. Many genetic diseases may be underlain by CNVs. However, because of the presence of their multiple copies, variability in copy numbers and the diploidy of the human genome, detailed genetic structure of CNVs cannot be readily studied by available techniques.

**Methodology/Principal Findings:**

Single sperm samples were used as the primary subjects for the study so that CNV haplotypes in the sperm donors could be studied individually. Forty-eight CNVs characterized in a previous study were analyzed using a microarray-based high-throughput genotyping method after multiplex amplification. Seventeen single nucleotide polymorphisms (SNPs) were also included as controls. Two single-base variants, either allelic or paralogous, could be discriminated for all markers. Microarray data were used to resolve SNP alleles and CNV haplotypes, to quantitatively assess the numbers and compositions of the paralogous segments in each CNV haplotype.

**Conclusions/Significance:**

This is the first study of the genetic structure of CNVs on a large scale. Resulting information may help understand evolution of the human genome, gain insight into many genetic processes, and discriminate between CNVs and SNPs. The highly sensitive high-throughput experimental system with haploid sperm samples as subjects may be used to facilitate detailed large-scale CNV analysis.

## Introduction

The human genome harbors extensive structural variation [1]–[4]. A copy number variant (CNV), is designated as a group of genomic DNA segments that are 1 kb or longer with a variable copy number and sharing >90% sequence identity [2]. Based on their structures, CNVs are classified as deletion, duplication, deletion and duplication, multi-allelic and complex [3]. CNVs have been shown abundant in the human genome [2]–[17]. Structure variation in CNVs such as gene sequence disruption and dosage variation may have significant impact on affected genes and gene expression [2], [13], [18]–[23], and may cause diseases [2], [21], [24]–[26].

Ability to study the genetic structures of CNVs may help understand the evolution of the human genome, gain insight into many genetic processes, and discriminate between CNVs and single nucleotide polymorphisms (SNPs). However, challenges in study of genetic structures of CNVs stem from multiple dimensions, including: (1) multiple CNV segments sharing a high degree of sequence identity; (2) similarity between allelic variants of SNPs and paralogous variants of CNVs; and (3) the diploidy of the human genome. Although some available technologies may be used for CNV detection, it is difficult to use these techniques to learn the genetic structures of CNVs. For detailed study, an experimental system capable of detecting minor sequence variation, discriminating between allelic variants and paralogous variants, determining CNV segment numbers of various kinds is needed.

**Figure 1:**
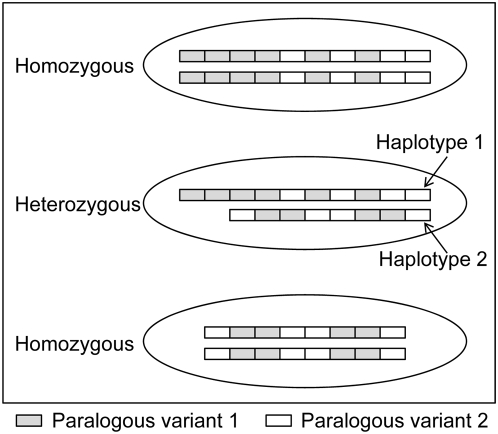
Schematic illustration of genotypes, haplotypes, and paralogous variants. Cells with three different genotypes comprised of two haplotypes are shown. The top and bottom cells are homozygous for either the longer or shorter haplotype, while the cell in the middle has both. Each haplotype has two paralogous variants that are distinguished by grey and white colors, and discriminated by analyzing a single-base substitution experimentally. Each variant may have zero to multiple copies.

In contrast to SNPs which have two allelic variants differing by a single base, a CNV may have more than two “alleles” that are actually haplotypes differing in the number of paralogous segments in the human population (Figure 1). In many cases, segments in each CNV haplotype may be subdivided into two paralogous variants distinguished by a single-base substitution similar to SNPs. Each variant may have zero to multiple copies. In this way, CNV haplotypes may be distinguished in their numbers and/or compositions of the paralogous segments. SNPs may be considered as single-segment CNVs and paralogous sequence variants (PSVs) [1], [5], [27] may be viewed as CNVs with identical segment numbers and compositions in their haplotypes. Since one can never prove a PSV a real PSV until the entire human population is analyzed, and PSVs and CNVs may be inter-convertible during evolution (see Results and Discussion sections), we consider PSVs also as CNVs in the present study.

In the study by Fredman *et al.* [28], CNVs were classified into three subgroups: (1) PSVs as defined above, (2) SNPs in duplicons (SIDs), each of which contains an SNP in a single paralogous segment, and (3) multi-site variants (MSVs). An MSV may be converted from an SID during evolution through the following process: the SNP-containing segment in an SID may have been duplicated and shuffled by various genetic events. Some of the duplicated segments may have been lost. As a result, the original SNP variants may be found at multiple sites, some of the original allelic variants may be no longer allelic. However, classification of CNVs into these subgroups may not be accurate and/or possible in reality. For example, a PSV may be detected in one ethnic group, but one or more haplotypes may be found in other ethnic groups (see Results and Discussion sections). If a CNV has only one copy for one paralogous variant and 5 copies for the other, it may be considered as an SID. However, experimentally, this cannot be distinguished from a CNV with 2 and 10 copies for the two paralogous variants, respectively unless the absolute number of CNV segments can be determined. On the other hand, the numbers of the CNV segments determined by most current approaches can only be relative. For these reasons, in the present publication we describe CNVs by their numbers of haplotypes among the analyzed samples and by the characteristics of these haplotypes. The classification information used by Fredman *et al.* is used only for reference and comparison.

The net genotyping signal for a CNV is from all individual segments with complex behavior [28]. Discrimination between SNPs and CNVs was a challenging issue. In attempt to circumvent these issues when appropriate methods are lacking, SNPs within annotated CNVs were often avoided in genotyping assay design [29], [30]. This is unfortunate, as it leaves these markers unused in many studies of candidate disease loci. Detection of CNVs has been facilitated in part by the recent development in microarray coverage and computer algorithms by industrial institutions such as Affymetrix and Illumina. However, knowledge about the presence of CNVs alone is far from knowing the detailed structures of these variations. To study CNVs in detail, one needs to understand their genetic structures, including their haplotypes in the human population, and number and composition of the paralogous variants in each haplotype. When the genetic structure becomes clear, CNVs can be easily discriminated from SNPs. The knowledge may help us understand human genome evolution, the role of CNVs in meiotic recombination, genetic stability of these sequences, and evolution of regions containing these sequences.

In the present study, haploid sperm samples were used as subjects. With these samples, allelic variants can be easily discriminated from paralogous variants because the former can only be detected from different sperm while the latter can be observed in a single sperm sample. Since CNV haplotypes are naturally segregated into different sperm cells during meiosis, genotype information from sperm samples is actually the information of individual haplotypes, making the study very simple in contrast to diploid cells for which genotype information is a mixture of information from two haplotypes. Furthermore, with single sperm, the haplotype composition in sperm donors and in the studied population, and paralogous variants in each haplotype can be easily studied.

## Results

Sixty-five markers characterized by Fredman *et al.* [28] were included in the present study. Of these markers, 48 were shown to be CNVs (MSVs, PSVs, or SIDs), and 17 were SNPs in unique regions and used as controls. First, we updated the genomic annotation of each marker with information from recent sequence databases (Table 1, also see Methods). Contrary to previous annotation, all CNVs mapped to multiple sites in the human genome, representing an improvement in coverage of CNVs in newer versions of the human genome assembly. All control SNPs mapped to unique genomic loci.

**Table 1:** dbSNP access numbers of 65 markers and classification by Fredman *et al.* [28].

MSV	PSV	SID	SNP
Rs394595	Rs633700	Rs1056119	Rs585664
Rs296349	Rs3019009	Rs375160	Rs623790
Rs505235	Rs1060021	Rs2651432	Rs713624
Rs746659	Rs624516	Rs406372	Rs632951
Rs2287968	Rs680347	Rs1363818	Rs94499
Rs2161510	Rs529820	Rs439825	Rs621287
Rs1042724	Rs2604079	Rs2641915	Rs1801018
Rs2698877	Rs2910550	Rs2903718	Rs2073449
Rs964055	Rs2960392	Rs440199	Rs1544210
Rs675597	Rs2388099	Rs1025356	Rs226005
Rs1057729	Rs2690640	Rs1754228	Rs589670
Rs2740083	Rs2931178	Rs879886	Rs710174
Rs2868008		Rs428259	Rs1188006
Rs2939843		Rs889206	Rs1545086
Rs2690641		Rs2194189	Rs2877021
Rs2781957		Rs2690645	Rs1230067
Rs2868007		Rs1059996	Rs597320
		Rs2461070	
		Rs595203	

Single base substitutions used in Fredman *et al.* were used to discriminate between the allelic (for SNPs) or paralogous (for CNVs) variants for all markers. We genotyped all 65 markers in 189 single sperm cells from 11 unrelated Northern European donors by microarray after multiplex PCR amplification of all marker sequences in a single tube. The natural logarithm of the ratio, Ln(R), between the signal intensities of the two colors representing the two variants of each marker was plotted against the sum of these two signal intensities. The genotypes of the sperm donors and segregating groups of the sperm from each donor were then determined using the scatter plot and statistically confirmed by the Student's *t*-test.

**Figure 2:**
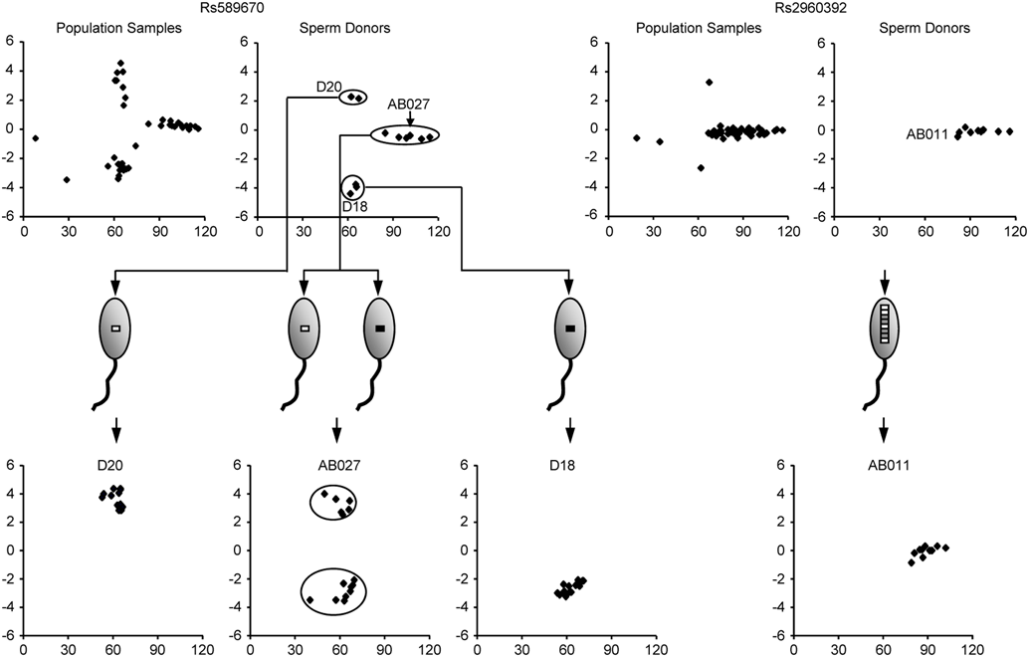
Correlation between genotypes of the donors and their sperm samples for SNP Rs589670 and CNV Rs2960392. Donor genotypes were determined using the corresponding semen samples. For all scatter plots: x-axis, the sum of the signal intensities/1,000 of the two colors, y-axis, Ln(R)s. Allelic variants of the SNP are diagrammed as light grey and black strips in the sperm heads, and CNV paralogous variants are indicated as white and darker grey strips.

The left panel of Figure 2 shows a typical result from SNP Rs589670. The signal intensities of the 11 sperm donors clearly clustered in three groups: two homozygous groups with Ln(R) values at either top or bottom of the plot, and a heterozygous group with Ln(R) values close to zero, as the signal intensities for the two SNP alleles were nearly equal. For each homozygous donor (for example, D20 and D18 in Figure 2), signal intensities of all his sperm fell into one cluster in the same range as the donor. For each heterozygous donor, two groups of sperm were observed (for example, AB027 in Figure 2) with the signal intensities matching those of the two groups of homozygous donors, clearly indicating segregation of the SNP alleles during spermatogenesis. In contrast, the signal intensities of PSV markers for all single sperm samples fell into the same range as their donor's (right panel, Figure 2). The mean Ln(R) values for five (42%) PSVs were close to zero, likely reflecting that the copy numbers of the two paralogous variants (indicated as grey and white strips in the sperm head in Figure 2) were equal or nearly equal. However, the other seven (58%) PSVs had Ln(R) values centered by a value deviated from zero, indicating a difference in the copy numbers of the two paralogous variants.

**Figure 3:**
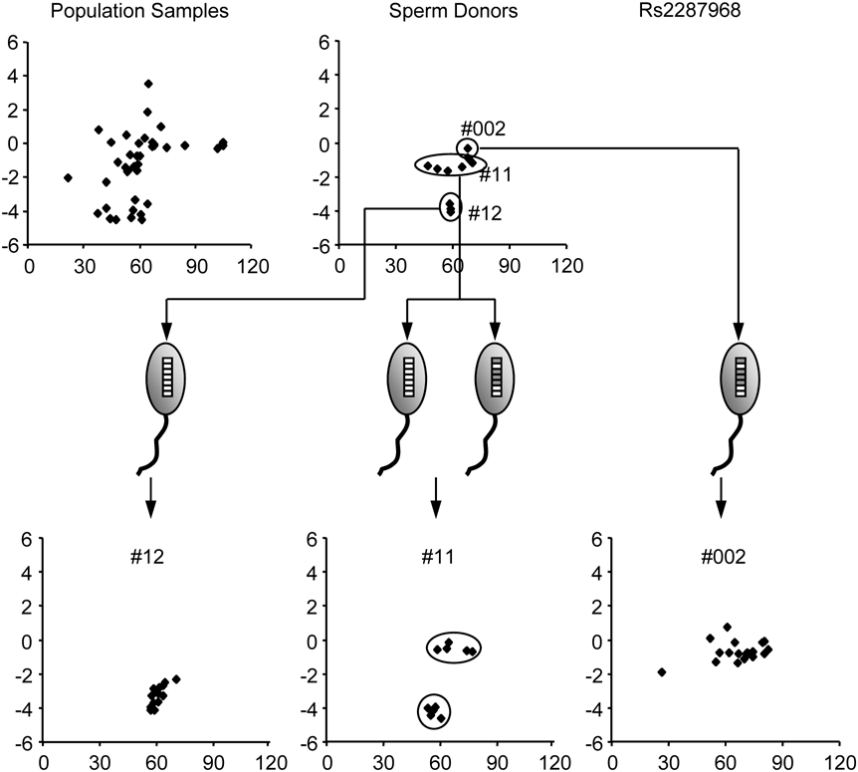
Correlation between genotypes of the donors and their sperm samples for CNV Rs2287968. Meanings of the graphics are the same as those in Figure 2.

When an SNP is located in a CNV, the signal intensities for the segregating alleles in the single sperm samples display characteristics different from those of SNPs in unique regions. As shown in Figure 3, the genotypes of the 11 donors for marker Rs2287968 can be clearly subdivided into three groups according to the genotypes of their sperm. The two groups represented by donors #12 and #002 clearly generated single groups of sperm indicating that these donors were homozygous for this marker. This was further confirmed by the sperm genotypes of the third group of donors represented by donor #11. Each donor (#11 is used for the illustration in Figure 3) in this group generated two groups of sperm with their Ln(R) values falling into the ranges of the two homozygous groups, indicating that the two “alleles” in these donors segregated into two groups of sperm during meiosis.

However, in contrast to the signals obtained from SNPs, the Ln(R) values of one of the two “alleles” for marker Rs2287968 fell into a range centered by −0.40. This cannot be explained by the behavior of an allele, and would be better understood as a CNV haplotype that was comprised of two paralogous variants (indicated as grey and white boxes in the sperm head in Figure 3).

To assess the copy numbers of the paralogous variants in the haplotypes of a sperm donor, we developed a mathematic model. As shown in Figure 1, each donor has two haplotypes for a CNV. The haplotypes could be identical (homozygous) or different (heterozygous) in the numbers and compositions of the two paralogous variants. We let the copy numbers of the segments in the two haplotypes be *h1* and *h2*, respectively. The two haplotypes have *m* and *n* copies for one of the paralogous variants, respectively, (the grey variant in Figure 1, for example). If all segments are amplified equally in each reaction, which has been shown in our high-throughput genotyping studies [31], [32], the fractions of the signal intensities for one of the two variants in the donors of the three different genotypes can be expressed, respectively as:
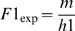
(1)

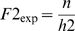
(2)

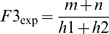
(3)


If we assign a group of values to the four variables, *h1*, *h2*, *m*, and *n*, the estimated values of these variables can be found using the Least-squared estimation method, i.e, the values that minimize the difference between the observed and the expected fractions under the set values using the following formula:
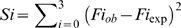
(4)


To find the minimum value of *Si*, a computer program was written. During looping of the four variables, *T* (which is the sum of *h1* and *h2*), *h1*, *m*, and *n* under a given value of *T_max_* which is an input number for the maximum number of *T* for computation, the 30 (or less when not available) least values of *Si* were recorded together with their corresponding *T*, *h1*, *m*, and *n* values. Because of the method is based on the expected and observed **fractions**, multiple solutions can be found for the variables with increments in proportion as *T* increases. We choose the group of values with *Si* immediately less than 1.00 as the “basic units.” They are so defined because the actual number of segments of each kind could be proportionally less or greater than the numbers of the basic unit. However, the ratios between the segment numbers of different variants in the haplotypes would remain constant.

Using this method, our estimate of the total copy number of the paralogous segments for Rs2287968 is 10 in heterozygous individuals, and 3, and 7 in the two haplotypes, respectively. Haplotype 1 has 0, and 3 segments for the two paralogous variants, and in haplotype 2 had 2 and 5, respectively. Haplotype 1 contained 0 segments for one paralogous variant and 3 for the other while haplotype 2 contained 2 segments for one variant and 5 for the other.

**Figure 4:**
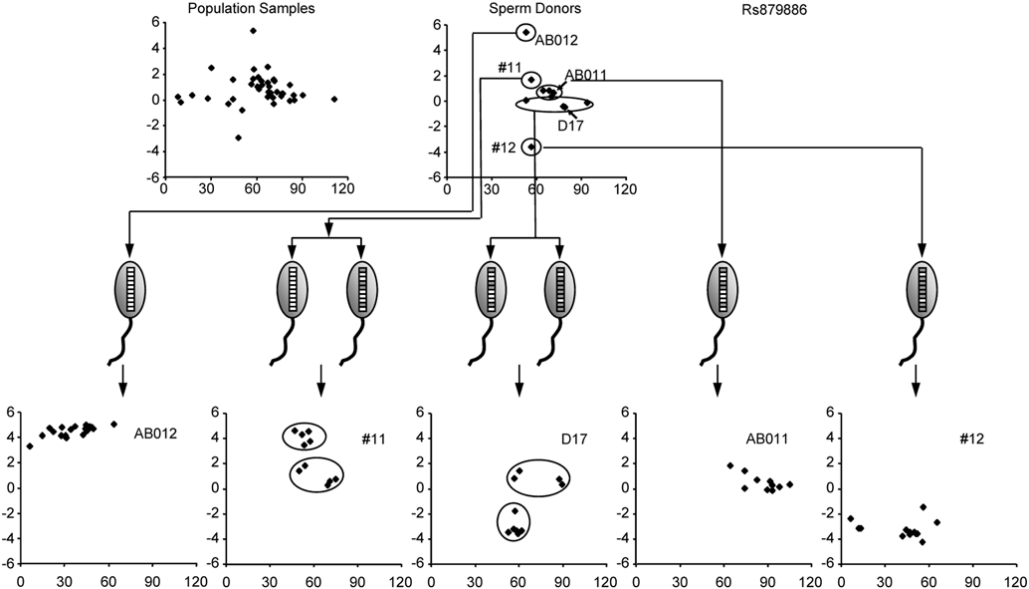
Correlation between genotypes of the donors and their sperm samples for CNV Rs879886. Meanings of the graphics are the same as those in Figure 2.

A CNV may have more than two haplotypes in the human population. When the number of haplotypes is large, the possible combinations of these haplotypes among human individuals could be very large. However, since individual haplotypes can be easily resolved by sperm analysis, CNV analysis is not complicated by the number of haplotypes or genotypes in the human population. The number and composition of each haplotype may be assessed using the above method. As shown in Figure 4, the Ln(R) values of the 11 donors for marker Rs879886 fell into five groups which can be further confirmed by their sperm genotypes. As shown, three donor groups containing donors AB011, #12 and AB012, respectively, were homozygous for three different haplotypes. The other two donor groups were heterozygous. It is clear that the genotype of each heterozygous donor was a combination of two out of the three haplotypes. Based on the signal intensities of these groups of donors and their sperm, the numbers of total segments of the basic unit in the three haplotypes were estimated as, 11, 7, and 5 with the numbers and compositions for the two paralogous variants of 11+0; 4+3; and 0+5, respectively.

A main feature of a CNV is all or some of their haplotypes with Ln(R) values falling between those of the SNP alleles. As shown in Figure 3, the Ln(R) for the “upper” haplotype of marker Rs2287968 has a mean value of −0.66, while the mean value for the “middle” haplotype of marker Rs879886 in Figure 4 is 0.67. These results reflect the differences in the ratios between the copy numbers of the paralogous variants in these haplotypes.

Based on the microarray data, all 17 SNPs were shown to be true SNPs. Marker Rs624516, which was previously described as a PSV, was excluded for further analysis because of its poor signal intensities from majority of the samples. The numbers of haplotypes resolved for the remaining 47 markers are listed in Table 2. As shown, only one haplotype was detected in the sperm samples for all 11 previously described PSVs. Only one haplotype was detected for 10 markers which were characterized as either SIDs or MSVs previously [28]. Two or three haplotypes could be resolved for each of the remaining 26 markers.

**Table 2:** Numbers of Haplotypes of the 48 CNVs.

Marker	Haplotypes Identified by			Previous Classification
	Sperm	Population	Total	
Rs2690640	1	0	1	PSV
Rs2910550	1	0	1	PSV
Rs529820	1	0	1	PSV
Rs2388099	1	1	2	PSV
Rs2604079	1	1	2	PSV
Rs2931178	1	1	2	PSV
Rs3019009	1	1	2	PSV
Rs633700	1	1	2	PSV
Rs680347	1	1	2	PSV
Rs1060021	1	2	3	PSV
Rs2960392	1	2	3	PSV
Rs624516	N/D	N/D	N/D	PSV
Rs1059996	1	1	2	SID
Rs2903718	1	1	2	SID
Rs375160	1	1	2	SID
Rs595203	1	1	2	SID
Rs2690645	1	2	3	SID
Rs1754228	2	0	2	SID
Rs2461070	2	0	2	SID
Rs439825	2	0	2	SID
Rs1363818	2	1	3	SID
Rs2194189	2	1	3	SID
Rs2641915	2	1	3	SID
Rs2651432	2	1	3	SID
Rs406372	2	1	3	SID
Rs428259	2	1	3	SID
Rs1025356	3	0	3	SID
Rs1056119	3	0	3	SID
Rs879886	3	0	3	SID
Rs889206	3	0	3	SID
Rs440199	3	1	4	SID
Rs2690641	1	0	1	MSV
Rs394595	1	0	1	MSV
Rs1057729	1	1	2	MSV
Rs2781957	1	1	2	MSV
Rs2868007	1	1	2	MSV
Rs505235	2	0	2	MSV
Rs746659	2	0	2	MSV
Rs964055	2	0	2	MSV
Rs1042724	2	1	3	MSV
Rs2161510	2	1	3	MSV
Rs2287968	2	1	3	MSV
Rs2868008	2	1	3	MSV
Rs2939843	2	1	3	MSV
Rs2698877	3	0	3	MSV
Rs296349	3	0	3	MSV
Rs675597	3	0	3	MSV
Rs2740083	3	1	4	MSV

To learn whether more haplotypes might be present in the human population, genotypes of additional 40 population samples from four ethnic groups (see Methods) were analyzed. The scatter plots were compared with the ones for the 11 sperm donors. As shown in Figure 3, for markers Rs2287968, we could clearly resolve two haplotypes among the 11 sperm donors. However, the Ln(R) values in the scatter plot for the 40 population samples cannot be all accounted by these two haplotypes. It is very likely that at least one more haplotypes were present among the 40 population samples. In this way, we determined the number of possible additional haplotypes among the 40 population samples. Results are summarized in Table 2.

As shown in Table 2, additional haplotypes were found for 29 (61.7%) of the 47 CNV markers among the 40 population samples, including eight (72.7%) of the 11 CNVs that were previously described as PSVs, indicating that CNVs are very genetically active and classification information based on genotyping a given number of individuals may not include all haplotypes in the human populations. A PSV detected in a small group of individuals may turn out to be an SID or MSV when more samples or samples from different ethnic groups are analyzed.

**Figure 5:**
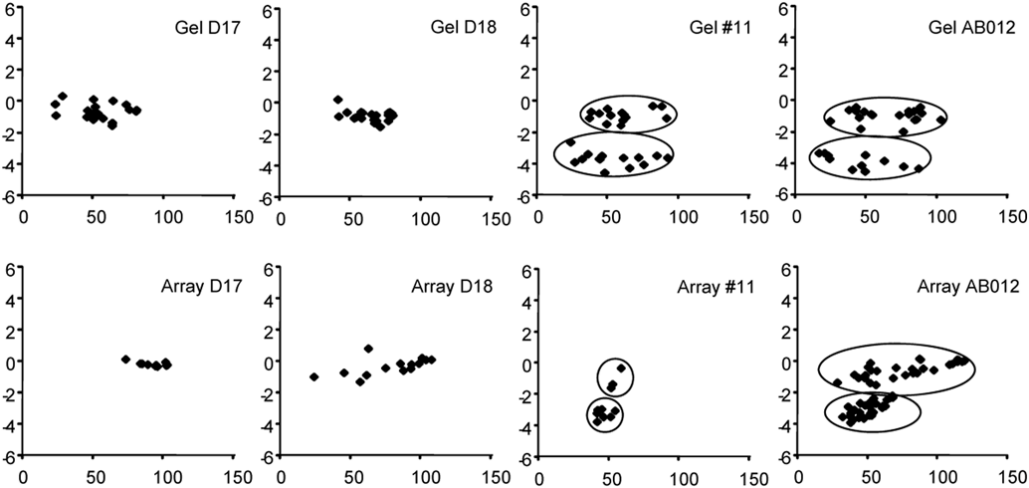
Comparison between data from microarray and gel electrophoresis. Meanings of the x- and y-axes in all scatter plots are the same as those in Figure 2.

The high degree of concordance between our experimental results and expectations based on meiotic segregation of the alleles/haplotypes has already demonstrated a high-level of accuracy and reliability of our system. To further prove its robustness, we used a different method and reanalyzed a subset of the samples and markers: four semen samples, D17, D18, #11 and AB012, and three markers, Rs2931178, Rs439825 and Rs440199 which were shown to have a single, two, and three haplotype(s) among the 11 sperm donors, respectively. No SNP marker was included in this round of study because the robustness of our system for genotyping of large panels of SNPs had been demonstrated in our previous publications [31], [32]. We prepared 317 single sperm using a manual procedure described previously [33]. The three CNV sequences were first co-amplified from each sperm sample followed by separate amplification using aliquots from the first round PCR products. The paralogous variants of the CNVs were resolved by digestion of the PCR products with appropriate restriction enzymes followed by gel electrophoresis. Signal intensities of gel bands were then determined. Genotypes of all markers and samples were consistent with those determined by microarray. Figure 5 shows a side-by-side comparison of the results from the two different methods for marker Rs439825.

## Discussion

Identification and characterization of CNVs represents a substantial technological challenge. Our simple initial strategy to identify sequences that are likely not unique in the human genome was by *in silico* analysis of publicly available human genomic sequence. However, this strategy is limited by the (incomplete) representation of CNVs in the human genome assembly. Recent discoveries of abundant CNVs [3], [4] argue that it is not possible to identify all CNVs in a population or patient material by trawling public databases and gain insight into the detailed genetic structures of CNVs. Thus, efficient experimental methods remain indispensable for this purpose.

PCR amplification in conjunction with gel electrophoresis might reveal CNVs if the lengths of the amplified sequences differ. Our screen of 65 markers with this method revealed CNV characteristics for only a small fraction (22.9%) of known CNVs. A clear pitfall with this method is its low specificity because nonspecific PCR products may be generated from other regions that are not target CNVs. In this case, no distinction could easily be made between CNV sequences and non-specific PCR amplicons based on size alone. Thus, gel electrophoresis of PCR products may not be an effective and reliable way to CNV screen.

The same set of 65 markers was also checked by genotyping DNA samples from the 40 human individuals from four different ethnic groups, a method commonly used to validate SNP to learn whether they are polymorphic or monomorphic, e.g. by TSC (http://snp.cshl.org/about/qa.shtml). We assigned CNV status to 60% (29/48) of previously identified CNVs correctly based on a 95% confidence interval for the possible maximum heterozygosity. All the CNVs identified by this method were also confirmed by single sperm analysis. Although this method is more sensitive than the gel assay, 40% of the known CNVs in our set remained unidentified.

Genotyping with a haploid genome is a very efficient approach to identifying markers residing in non-unique sequence and to gain insight into the detailed genetic structures of CNVs. The rational is straightforward. With this method, true allelic difference can only be observed between different sperm samples, while paralogous differences can be seen in the same individual gametes. The present study is the first application of single sperm analysis in conjunction with our recently developed high-throughput genotyping system to the analysis of the detailed genetic structures of CNVs. Single sperm analysis allowed us to separate haplotypes of each sperm donor so that the complications associated with diploid genome were eliminated. By quantitative analysis of signals from the two paralogous variants of each haplotype, we were able to assess the number of haplotypes of each CNV among the studied samples, the relative numbers of paralogous segments in each haplotype, and the composition of the haplotypes in term of the paralogous variants.

Complete hydatidiform moles (CHMs) are fully homozygous genomes, and were used for CNV study previously [28]. Compared with CHMs, sperm samples may be used to study genotypes identified in any human male from whom semen samples can be collected, while CHM can only be used to analyze genotypes identified among these samples. In contrast to the medical restriction of CHM sample application, single sperm analysis offers a great advantage in sample availability. Semen samples are easy to collect, can be stored for many years and retain good quality for genetic analysis while passage of CHM cells may have to subject to accumulation of genetic alterations. Compare to the CHM samples that can be reused many times, a single sperm can only be used once. Such a limitation is not a concern for the described study. The reusability of the sperm samples can be viewed in two aspects. Firstly, since our approach is based on the fractions of sperm of each type, not individual sperm, it is not critical whether we can reuse single sperm samples. Since a semen sample contains practically an unlimited number of sperm, it can be used for actually an unlimited number of assays given that our system is so sensitive that one sperm can be used for analyzing >1,000 sequences. Secondly, we have shown that a single sperm may be subjected to whole genome amplification. For >1,000 SNPs, a small aliquot (2 µl) of the amplified product would be sufficient for each assay (Cui & Li, unpublished data). In this way, sperm samples may be reused and thousands of markers in each sperm may be analyzed.

Results from the present study are not all consistent with those reported by Fredman et al. [28]. The most likely reason, as mentioned above, could be the difference between the samples used in these studies. A CNV haplotype could be predominantly present in some ethnic groups but not in others. Small sample size may also cause bias in the haplotype frequencies. Another reason for the discrepancy could be difference in resolution of these methods. Using sperm analysis, heterozygous genotypes consisting of different CNV haplotypes can be precisely resolved. For example, for the samples in Figure 4, we identified five genotypes consisting of different combinations of only three haplotypes. Therefore, our single sperm method may significantly reduce the complexity of genotypes, which is difficult with other methods. The amount of work involved in typing multiple sperm samples for each sperm donor is offset by using microarrays with which thousands of markers can be analyzed simultaneously making the sperm method a highly reliable and efficient method.

With emerging ability to assay single sperm with quantitative results, such as in an emulsion PCR, we could begin assess the frequency of duplication and deletion events in single molecules, giving further clues to which regions are prone to such CNVs, and to which extent they get transmitted across generations.

In summary, we demonstrated that single-sperm typing is a very efficient and reliable way to learn great details of genetic structures of CNVs in the human genome, and may allow us to study genetic events occurring in the chromosomal regions containing these sequences.

## Methods

### Genetic Markers and DNA/Sperm Samples

Seventeen MSVs, 12 PSVs, 19 SIDs and 17 SNPs in unique sequences described in Fredman *et al.* [28] (Table 1) were selected for study. Forty genomic DNA samples from four ethnic groups, African American, American Indian, Caucasian, and Chinese (10 samples each), were purchased from the Coriell Institute for Medical Research (Camden, NJ). Semen samples from 11 donors were used. These samples were collected for previous projects by an infertility test laboratory and sent to us anonymously. Because they were pathological remains, our Internal Review Board approved their status as exempt from regulations. Because these samples were not specifically collected for the present study, the present study using these samples should be considered as no involvement of human subjects according to the human subjects regulations 45 CFR Part 46 of the U.S. Department of Health and Human Services. All donors were shown to be normal in fertility.

Single sperm samples were prepared by flow cytometry. Sperm DNA was released and prepared ready for multiplex amplification as described previously [33]. We used 10 to 20 sperm from each donor except one, AB012, for whom 62 sperm were analyzed. We compared the validation rates for assays using 10 sperm and those using more than 10 sperm and found that the number of tested sperm had no significant effect on the determination rate (*p* = 0.95, T test; and *p* = 0.65, χ^2^ test).

### Overlap with Repetitive Sequences

We performed in silico search for evidence of non-unique character for all PCR amplicons by three different methods: (1) BLAT search against a non-redundant representation of the human genome [34]; (2) BLAST search against redundant human genome sequences [35], [36]; and (3) screen for interspersed repeats and low complexity DNA sequences using RepeatMasker Version: 3.0.2 (http://www.repeatmasker.org), all using standard parameters.

### Multiplex Amplification and Microarray Analysis

The genomic DNA samples and DNA from single sperm were subject to multiplex amplification followed by microarray analysis as described elsewhere [32]. Briefly, for each sample, the polymorphic sequences of each multiplex group were amplified by multiplex PCR which was performed in 30 µl of PCR mix containing 1×PCR buffer (50 mM KCl, 100 mM Tris-HCl at pH 8.3, 1.5 mM MgCl_2_, and 100 µg/ml gelatin), four dNTPs (200 µm each; Invitrogen), primers (20 nM each) for all 65 markers included in the present study, 6 units of HotStart *Taq* DNA polymerase (QIAGEN, Valencia, CA, USA), and 5 ng of genomic DNA or DNA released from a single sperm. The samples were first heated to 94°C for 15 min to activate the *Taq* DNA polymerase followed by 40 PCR cycles. Each PCR cycle consisted of 40 sec at 94°C for denaturation and 2 min at 55°C followed by 5 min of ramping from 55°C to 70°C for annealing and extension. A final extension step was carried out at 72°C for 3 min at the end of the 40th cycle. PCR amplifications were performed with thermal cyclers capable of ramping as slow as 0.01°C/sec, including the PTC100 Programmable Thermal Controller (MJ Research), T3 Thermocycler (Biometra), and PxE Thermal Cycler (Thermo Electron). Single-stranded DNA (ssDNA) was generated by using a mixture of one primer from each pair and a small amount (1–2 µl) of the multiplex PCR product under the same conditions used for multiplex PCR. The resulting ssDNA was hybridized to an oligonucleotide probe microarray on a glass slide. The probes were designed in such a way that their 3′-ends were immediately next to the polymorphic sites in the ssDNAs, and were specifically extended with fluorescent-labeled dideoxyribonucleotides (ddNTPs) in the presence of Sequenase [37]. The intensities of different fluorescent colors were obtained after scanning the microarray and digitizing the resulting image. Genotypes were determined by using the Accutyping [38] software. Semen samples were genotyped using each of the two DNA strands as templates, respectively. Markers that did not have identical genotypes for both strands were labeled inconsistent.

### Genetic analysis by restriction enzyme digestion and gel electrophoresis after PCR amplification

Restriction enzymes, Fun4HI and DdeI, were purchased from the New England Biolabs (Ipswich, MA, USA). Single sperm samples were prepared manually using a procedure described previously [33]. Three CNVs, Rs2931178, Rs439825 and Rs440199, were co-amplified by multiplex PCR from lysed single sperm samples. Three 2-µl aliquots from each PCR product were re-amplified separately using primers for the three CNV markers, respectively. PCR conditions were the same as described above, except for 25 cycles for the second round of amplification. From each final PCR product, a 4-µl aliquot was digested with 2 units of the respective restriction enzyme for 1 hr at 37°C. The digested products were analyzed using 10% PAGE. Gels were stained with ethidium bromide and imaged using a gel documentation system, GelDoc-It™ (UVP, Upland, CA, USA). Gel bands were digitized and signal intensities in the gel bands were determined using the software VisionWorks®LS purchased with the gel documentation system.
